# Rare inherited kidney diseases: an evolving field in Nephrology

**DOI:** 10.1590/2175-8239-JBN-2018-0217

**Published:** 2020-03-20

**Authors:** Mariana Faucz Munhoz da Cunha, Gabriela Sevignani, Giovana Memari Pavanelli, Mauricio de Carvalho, Fellype Carvalho Barreto

**Affiliations:** 1 Universidade Federal do Paraná, Departamento de Pediatria, Serviço de Nefrologia Pediátrica, Curitiba, PR, Brasil.; 2 Hospital Pequeno Príncipe, Serviço de Nefrologia Pediátrica, Curitiba, PR, Brasil.; 3 Universidade Federal do Paraná, Departamento de Clínica Médica, Curitiba, PR, Brasil.; 4 Universidade Federal do Paraná, Departamento de Clínica Médica, Serviço de Nefrologia, Curitiba, PR, Brasil.

**Keywords:** Genetic Diseases, Inborn, Kidney Diseases, Glomerulosclerosis, Focal Segmental, Fabry Disease, Tuberous Sclerosis, Doenças Genéticas Inatas, Nefropatias, Glomerulosclerose Segmentar e Focal, Doença de Fabry, Esclerose Tuberosa

## Abstract

There are more than 150 different rare genetic kidney diseases. They can be classified according to diagnostic findings as (i) disorders of growth and structure, (ii) glomerular diseases, (iii) tubular, and (iv) metabolic diseases. In recent years, there has been a shift of paradigm in this field. Molecular testing has become more accessible, our understanding of the underlying pathophysiologic mechanisms of these diseases has evolved, and new therapeutic strategies have become more available. Therefore, the role of nephrologists has progressively shifted from a mere spectator to an active player, part of a multidisciplinary team in the diagnosis and treatment of these disorders. This article provides an overview of the recent advances in rare hereditary kidney disorders by discussing the genetic aspects, clinical manifestations, diagnostic, and therapeutic approaches of some of these disorders, named familial focal and segmental glomerulosclerosis, tuberous sclerosis complex, Fabry nephropathy, and MYH-9 related disorder.

## Resumo

As doenças renais genéticas raras compreendem mais de 150 desordens. Elas podem ser classificadas segundo achados diagnósticos como (i) distúrbios do crescimento e estrutura, (ii) doenças glomerulares, (iii) tubulares e (iv) metabólicas. Nos últimos anos, houve uma mudança de paradigma nesse campo. Os testes moleculares tornaram-se mais acessíveis, nossa compreensão sobre os mecanismos fisiopatológicos subjacentes a essas doenças evoluiu e novas estratégias terapêuticas foram propostas. Portanto, o papel do nefrologista mudou progressivamente de mero espectador a participante ativo, parte de uma equipe multidisciplinar, no diagnóstico e tratamento desses distúrbios. O presente artigo oferece um panorama geral dos recentes avanços a respeito dos distúrbios renais hereditários raros, discutindo aspectos genéticos, manifestações clínicas e abordagens diagnósticas e terapêuticas de alguns desses distúrbios, mais especificamente a glomeruloesclerose segmentar e focal familiar, complexo da esclerose tuberosa, nefropatia de Fabry e doença relacionada ao MYH9.

## Introduction

There is no consensual definition for rare diseases and, although based upon the prevalence, the threshold varies across the globe. Europe and Japan define a rare disease as one affecting less than 1 in 2,000 and 1 in 2,500 individuals, respectively; while the USA consider a disease as rare when it affects fewer than 200,000 people around the country 1. Brazil follows the World Health Organization (WHO) definition, which considers rare a disease that affects less than 65 per 100,000 individuals. Data from INTERFARMA (Pharmaceutical Research Industry Association) estimates that there are approximately 13 million people affected by rare diseases in Brazil 2 ^,^ 3. The majority of rare diseases have genetic origin and half of them affect children.

Rare diseases have peculiar characteristics that challenge both patients and physicians. From the patient point of view, these diseases can cause many physical and mental disabilities, affecting life expectancy and quality of life, besides imposing a huge emotional burden on the families 1. The diagnostic delay is another important issue, reflecting the paucity of medical information about rare diseases. For instance, a European study of eight rare diseases reported an elapsed time up to 30 years from the first symptoms until the diagnosis in 25% of the patients and 40% of incorrect diagnosis 4. Furthermore, the widespread availability of molecular tests and the development of new therapies along the past decades added discussion and uncertainties to the medical practice. Doubts about treatment indication, legal rights to access high cost medications and its impact either on public or private health systems have raised concerns. When treatment should be initiated, who should be treated, when treatment should be stopped are recurrent questions in this area.

Most of the genetic kidney diseases are rare. Together, they comprise more than 150 different disorders 5. Monogenic causes can be identified in about 20% of patients with manifestation of chronic kidney disease (CKD) before 25 years old and in up to 10% of adults receiving renal replacement therapy 2 ^,^ 5. These diseases can be grouped according to diagnostic findings (Table 1) as: (i) disorders of growth and structure; (ii) glomerular diseases; (iii) and tubular diseases; and (iv) metabolic diseases 6. Among all these several different disorders, four of them were chosen to be discussed in this article, taking into account their prevalence and clinical importance, and the authors’ experience: focal segmental glomerulosclerosis (FSGS) and MYH-9 nephropathy (both causes of glomerular diseases), tuberous sclerosis (a ciliopathy), and Fabry nephropathy (a metabolic disease).

**Table 1:** Examples of genetic kidney diseases grouped according to the main diagnostic findings.

Diseases		Inheritance	Main clinical findings
Growth and structural abnormalities	CAKUT	AD	Renal agenesis/dysplasia, vesicoureteral reflux, posterior urethral valve
	ADPKD *	AD	Polycystic kidneys, liver and pancreatic cysts, cerebral aneurism, ESKD
	Bardet-biedel *	AR	Retinal dystrophy, obesity, mental retardation, limbs defects, renal abnormalities, ESKD
	TSC *	AD	Renal angiomyolipomas, angiofibromas, ungula fibromas, seizures, ESKD, etc.
Glomerular diseases	FSGS	AD	Late-onset SRNS, ESKD
		AR	Early-onset SRNS, ESKD
	Alport syndrome	X-linked	familial hematuria, lenticonus, neurosensorial deafness, ESKD
		AR	
		AD	
Tubular diseases	Bartter syndrome	AR	hypokalemic alkalosis, hypercalciuria, polyuria, growth retardation
	Gitelman syndrome	AR	hypokalemic alkalosis, hypocalciuria, hypomagnesemia
	cystinuria	AR	Cystin calculi, ESKD
Metabolic diseases	nephropathic cystinosis**	AR	Fanconi syndrome, photophobia, hypothyroidism, ESKD
	Fabry disease**	X-linked	Angiokeratomas, proteinuria, stroke, myocardial infarction, ESKD
	primary oxaluria	AR	Kidney and bladder stones, ESKD

Abbreviations: ADPKD: autosomal dominant polycystic kidney disease; AD: autosomal dominant, AR: autosomal recessive; CAKUT: congenital abnormalities of the kidney and urinary tract; ESKD: end-stage kidney disease; FSGS: focal and segmental glomerulosclerosis; SRNS: steroid resistant nephrotic syndrome; TSC: Tuberous Sclerosis Complex.

Footnote:

* ciliopathies.

** Some metabolic diseases, such as Fabry disease and nephropathic cystinosis, may lead to both glomerular and tubular injury.

### Genetic focal segmental glomerulosclerosis

Focal segmental glomerulosclerosis (FSGS) is not a single diagnosis but a group of clinical-pathological syndromes with the same pattern of histological glomerular injury 7. Despite of being difficult to establish the real incidence of FSGS, its average rate is around 2 patients per million population per year, with considerable variability depending on many factors, including access to diagnosis and racial/ethnic aspects. FSGS is the leading glomerular cause of end stage renal disease (ESRD) in USA adults and responsible for 15% of children requiring renal replacement therapy (RRT) 8. FSGS may be classified as primary, so-called idiopathic, secondary to an underlying disease, or genetic 8 ^,^ 9. Despite of its rarity, the familial forms of FSGS represent a significant range of patients with steroid-resistant nephrotic syndrome 10.

More than 50 genes related to FSGS have been described so far, most of them related to function or structure of podocytes. The identification of these genes and the understanding of the role of the mutation type in physiopathology and genotype/phenotype characterization have redefined diagnosis, treatment, and prognosis of nephrotic syndrome 11 ^,^ 12. The genetic causes of FSGS may present as sporadic or familial disease, with autosomal dominant, autosomal recessive, X-linked, or mitochondrial (matrilineal) inheritance patterns. Clinical manifestations follow different patterns. Several disorders affect early glomerular development and present during infancy or even prenatally, whereas others express nephrotic syndrome in adulthood. Some of the gene mutations (Table 2) that cause childhood and adulthood disease presentation are discussed below. A more detailed review of gene mutations related to FSGS may be found elsewhere 12.

**Table 2:** Example of genetic FSGS clinical characteristics according to mutated genes.

Gene inheritance	Protein	Phenotype	Resistance to immunosuppression
*NPHS1*	Nephrin	Finnish type congenital NS, rarely childhood and adulthood FSGS	Yes^#^
AR			
*NPHS2*	Podocin	appears in early childhood, but may also appear in adolescence or adulthood	Yes^#^
AR			
*MYO1E*	Nonmuscle myosin 1e	Familial childhood-onset (usually 1 – 9 years old)	Partial response to cyclosporin was reported
AR			
*ACTN4*	Alpha-actinin 4	Familial or sporadic adult-onset, early progression to CKD	Yes
AD			
*TRPC6*	Transient receptor potential cation channel 6	Sporadic adult-onset NS, early progression to CKD	Partial response to cyclosporin was reported
AD			
*INF2*	Inverted formin 2	Adolescence and adult-onset, association with Charcot-Marie-Tooth	Yes
AD			
*ARHGAP24*	RhoGTPasa activating protein 24	Adolescence-onset FSGS	Yes
AR			
*PTPRO*	Protein tyrosin phosphatase receptor type 0	Childhood-onset FSGS	Yes*
AR			
*CD2AP*	CD2 associated protein	Childhood-onset FSGS	Yes
*AD, rarely AR*			
*COQ2*	Coenzyme Q2 4hydroxybenzoate polyprenyl transferase	Childhood-onset FSGS, encephalopathy	Yes.
AR			May respond to coenzyme Q10
*COQ6*	Coenzyme Q6 4monoxygenase	Childhood-onset FSGS, sensorineural deafness	Yes.
AR			May respond to coenzyme Q10

Abbreviations: AD: autosomal dominant; AR: autosomal recessive; CKD: chronic kidney disease; FSGS: focal and segmental glomerulosclerosis; NS: nephrotic syndrome.

Footnote:

# response in heterozygous mutation has been reported;

* partial response has been reported.

The majority of these mutations follows an autosomal recessive pattern of inheritance and is mainly associated with *NPHS1* and *NPHS2* gene mutations, which encode nephrin and podocin, respectively, being both podocytes transmembrane proteins of the slit diaphragm 13. The loss of integrity of the glomerular filtration barrier results in early onset of nephrotic syndrome, which may be as early as in the first year of life, and in rapid progression to ESRD.

*NPHS1* gene mutations cause Finnish type nephrotic syndrome, the prototype for congenital nephrotic syndrome. More than 200 mutations in *NPHS1* have been identified, although most cases (90%) have Fin-major and Fin-minor mutations. Patients develop massive proteinuria starting within 3 months of life and do not respond to specific therapy for reducing proteinuria. Complications and mortality rates are high. Bilateral nephrectomy and peritoneal dialysis followed by kidney transplantation have been considered the treatment of choice 14.

Patients with *NPHS2* gene mutations usually present with nephrotic syndrome before 6 years old. The age at disease onset seems to depend on the type of mutation. Some patients may develop late onset forms of FSGS, starting at adolescence or early adulthood. Most cases do not respond to standard steroid therapy, the rate of extra renal complications of nephrotic syndrome is high, and the evolution to ESRD is generally fast 10.

Recently, mutations in the *MYO1E* gene, which encodes a non-muscle class I myosin related to podocyte cytoskeleton, were identified as a cause of autosomal recessive FSGS. Clinical manifestations, which appear between 1 and 9 years of age, are characterized by nephrotic range proteinuria or nephrotic syndrome, microscopic hematuria and early progression to CKD. Partial remission can be achieved using steroids, ACE inhibitors, and cyclosporine 7.

Autosomal dominant forms of FSGS frequently present during adolescence or adulthood 13. Mutations in the *ACTN4* gene have been recognized as culprits of autosomal dominant FSGS. This protein is part of the podocyte cytoskeleton and its abnormalities result in derangement of the architecture of the foot processes. The patients usually develop proteinuria during adulthood with slow progression to advanced CKD. Several mutations in *TRPC6* gene have also been associated to autosomal dominant FSGS. This gene encodes cationic channels and its mutation leads to increased calcium influx, resulting in glomerular dysfunction. The onset of proteinuria is usually during the third and fourth decades of life and up to 60% of patients progress to ESRD in 10 years 15. Another autosomal dominant form of the disease was recently associated with mutations in *INF2*, a protein that regulates actin polymerization. Clinical manifestations comprise appearance of mild proteinuria during adolescence or adulthood, microscopic hematuria, hypertension, and progression to ESRD 16. Interestingly, mutations in *INF2* gene seems to be related to FSGS associated with Charcot-Marie-Tooth neuropathy, one of the most frequent peripheral motor and sensory neuropathies and the most common inherited neuromuscular disorder 17.

Other gene mutations, for instance in *ARHGAP24, PTPRO*, and *CD2AP* genes, have been associated to familial FSGS 18. Furthermore, polymorphisms of the *APOL1* gene have been associated to greater risk of FSGS in African American individuals 19.

Molecular testing has not been routinely recommended for adult patients with FSGS, even when associated with steroid resistance. Specific mutations have been detected in less than 15% of cases when there is no history of familial FSGS 20. However, the identification of a genetic form of FSGF can be very helpful. It is a tool to guide treatment, avoiding the overuse and side effects of steroids and immunosuppressive medications 11. The low recurrence rate after kidney transplantation seen in patients with genetic forms of FSGS is also a very important information as the possibility of genetic counseling regarding living donor selection and chances of giving birth to an affected child 11.

Some findings may alert the nephrologist to the necessity of genetic investigation: (i) young age of disease presentation; (ii) familial nephropathy background, such as pediatric or adult patients with familiar history of ESRD or nephrotic syndrome; and (iii) children with FSGS not responding to conventional immunosuppressive treatment. In the near future, increased availability and lower cost of molecular tests may facilitate the genetic investigation

There is no specific therapy for genetic forms of FSGS, although the progress in understanding podocyte physiology opens new targets of treatment, as modulation of TRPC5 and TRPC6 channel activity 21. Still, some studies have shown resolution of proteinuria and a slower progression of chronic kidney disease with high-dose CoQ_10_ supplementation in mitochondrial disorders, due to COQ2 gene mutations, which present with nephrotic syndrome in children 22.

Blockade of the renin-angiotensin system has been recommended for children and adults with familial nephrotic syndrome. No clinical trials have provided any strong evidence on the efficacy of immunosuppressive therapy. Some studies have suggested that these medications could slow down the progression to ESRD in some patients, though. Kidney transplantation is considered a good option, since recurrence rates are very low 18.

### Tuberous Sclerosis Complex

Tuberous Sclerosis Complex (TSC) is a rare genetic disorder characterized by the presence of multiple benign tumors in several different organs and systems, such as brain, kidneys, heart, lungs, eyes, liver, and skin. It is caused by mutations in *TSC1*, located at chromosome 9, or *TSC2*, located at chromosome 16, genes that encode hamartin and tuberin, respectively. The hamartin-tuberin complex regulates cell growth and proliferation by inhibiting the mammalian-target-of-rapamycin (mTOR). Mutations in these genes result in activation of mTOR leading to uncontrolled cell proliferation and development of multiple organs hamartomas 23. The estimated birth incidence is 1:5,000 to 10,000 24 ^,^ 25.

The pattern of inheritance is autosomal dominant disease, with an almost complete penetrance. The prevalence of *TSC2* and *TSC1* mutations is similar in familial cases, while *TSC2* mutations are more frequent in sporadic cases. *De novo* mutations represent 80% of cases of TS. In 10-15% of the cases the mutation is not identified, particularly when it occurs in noncoding regions or due to mosaicism 23 ^,^ 25.

There is a wide variety of phenotypes of TSC in terms of clinical manifestations, age of onset, and number and severity of lesions. Even though no genotype-phenotype correlation has been established, *TSC2* mutations have been associated with more severe manifestations 26. Neurologic, dermatologic, and renal features are the most common findings. There is an increased risk for malignancy particularly in the brain, the kidneys, and soft tissues, in both children and adults 23.

The diagnosis of TSC is based on clinical criteria (Table 3). The presence of two major features or one major plus two minor features is required for the diagnosis. The age of onset of clinical manifestations is variable. Rhabdomyomas develop during fetal life while ungula fibromas in adolescence and adulthood 23 ^,^ 25. Although molecular testing is not necessary for the diagnosis, it is useful for genetic counseling and to confirm suspected TSC in patients who do not fulfill the diagnostic criteria. Together with medical history and careful dermatological and neurological examination, imaging studies may be indicated to identify the lesions and to evaluate its progression. Sometimes kidney biopsy may be required.

**Table 3:** Tuberous sclerosis complex: diagnostic criteria.

Major features
Angiofibromas (≥3) or fibrous cephalic plaque
Ungual fibromas (≥2)
Hypomelanotic macules (≥3, at least 5-mm diameter)
Shagreen patch
Multiple retinal hamartoma
Cortical dysplasia
Subependymal nodules
Subependymal giant cell astrocytoma
Renal angiomyolipomas (≥2)*
Cardiac rhabdomyoma
Lymphangioleiomyomatosis *
Minor features
Dental enamel pits ( > 3)
Intraoral fibromas (≥ 2)
Nonrenal hamartoma
Retinal achromic patch
Multiple renal cysts
"Confetti" skin lesions

* The single presence of renal angiomyolipomas and lymphangioleiomyomatosis does not meet criteria for the diagnosis of tuberous sclerosis complex.

Neurologic lesions are a common finding in TS, including hamartomas and subependymal nodules. Seizures are present in up to 90% of individuals. Autism, cognitive impairment, and behavioral problems are other neurological manifestations of the disease and present with wide variability 23 ^,^ 25. Almost all patients have skin lesions. Hypomelanotic macules are observed in around 90% of cases of TSC. Shagreen patches, angiofibromas, also called fibroadenomas, and fibrous plaque on forehead (sometimes the most readily recognized manifestation) are the most frequent dermatological findings 23.

**Figure 1:**
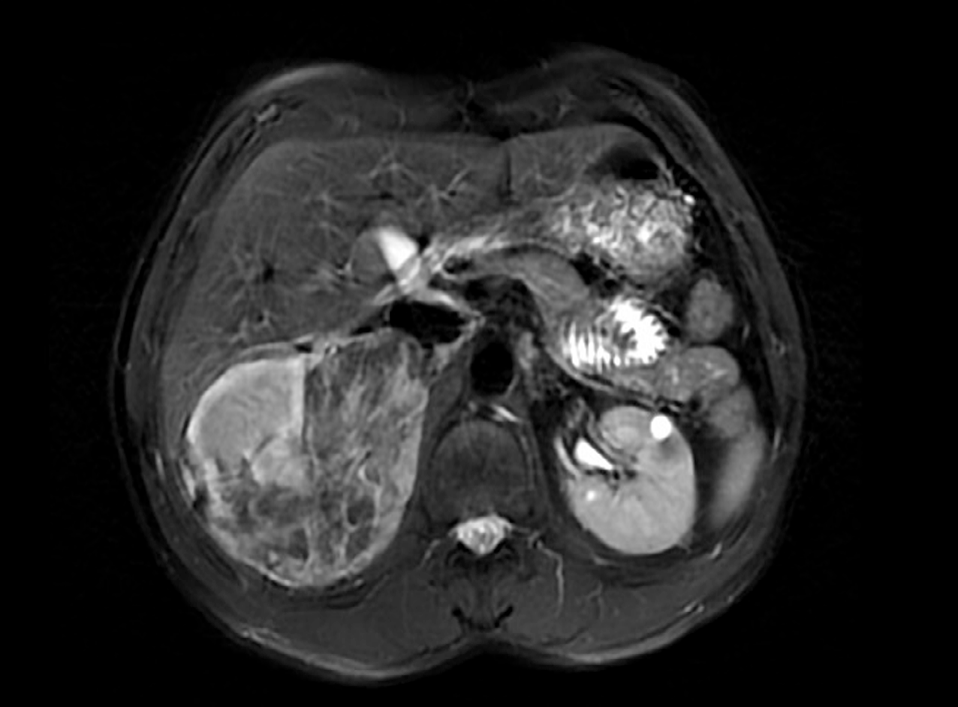
Bilateral multiple angiomyolipomas seen by magnetic resonance imaging in a 35-year-old male patient with tuberous sclerosis complex.

Renal manifestations occur in 50 to 80% of TSC patients and are associated with high morbidity and mortality. Their prevalence seems to increase with age, being 10 years old the mean age of presentation 27 ^,^ 28. Angiomyolipomas are the most frequent renal manifestation, reaching up to 80% of patients (Figure 1). They are benign lesions, usually multiple and bilateral, that tend to increase in size and number with age. They may cause bleeding, because of their high vascularity, in addition to pain, mass effect, urine flow obstruction, and renal parenchyma distortion. Renal cystic disease has been associated with TSC in 50% of patients, presenting with single or multiple cysts. The *TSC2* gene is contiguous to *PKD1* gene and deletions in both genes, called contiguous genetic syndrome (CGS), lead to autosomal dominant policystic kidney disease resulting in hypertension and renal dysfunction. Patients with TSC have the same incidence (2-3%) of renal cell carcinoma of the general population, but it usually appears earlier in the former. Importantly, it may be hard to differentiate it from fat-poor angiomyolipomas. Although contrast-enhanced magnetic resonance imaging or computerized tomography may help distinguishing them, sometimes biopsy might be required 29. CKD may develop by several mechanisms, as encroachment of renal parenchyma by angiomyolipomas, loss of renal parenchyma due to embolization or nephrectomy, contiguous gene syndrome *TSC2/PKD1*, interstitial fibrosis, and glomerulocystic kidney disease. ESRD affects 1 in 100 patients and renal complications are the main cause of death in TSC population 23.

The management of TSC has been mainly directed to the clinical manifestations. More recently, therapy with mTOR inhibitor, such as sirolimus or everolimus, has been proposed to target the underlying pathogenic dysregulation of mTORC1 signaling present in TSC. Clinical trials have reported that everolimus is capable of reducing angiomyolipoma size and the risk of bleeding with a relatively safe profile 30. For instance, sirolimus reduced angiomyolipoma lesion size by ~30% during 12 months of treatment 31. The clinical trial EXIST-2 was the first to evaluate everolimus for the management of renal angiomyolipomas. The response rate (defined as a ≥50% reduction in angiomyolipoma volume) after ~8 months of treatment was 42% for the everolimus-treated patients versus 0% for patients in the placebo arm 32. Importantly, the response rate was time-dependent. It increased to 54% in patients treated for a median of 29 months and increased further to 58% after 4 years 32. Subsequent post-hoc analysis of the EXIST-1 demonstrated a treatment response of 75% among everolimus-treated pediatric patients 33.

Some commonly reported adverse events associated with mTOR inhibitor therapy were: stomatitis/mucositis, infections, hypophosphatemia, hypertriglyceridemia, hypercholesterolemia, hematologic abnormalities (anemia, mild neutropenia, and leukopenia), and proteinuria 30 ^,^ 32 ^,^ 34. International guidelines have recommended mTOR inhibitors as first-line therapy for asymptomatic growing angiomyolipomas of 3 cm in diameter 35. Importantly, it has been postulated that mTOR inhibition may potentially worsen the progression of CKD 36 and the long-term effects of this therapy are not yet known. Currently, it has been advocated that surgical interventions, being kidney-sparing resection or embolization the preferred ones, should be reserved for exceptional cases, such as to prevent bleedings in those with more than 4 cm in diameter, high vascularity, big aneurysms, or suspicion of malignancy, and in the presence of acute hemorrhage 29 ^,^ 37.

### MYH9-related disease

*MYH-9* related disease (*MYH9-*RD) is a rare genetic disorder caused by mutations in the *MYH-9* gene, which encodes the myosin heavy chain 9 component of non-muscle myosin IIA (NMMHC-IIA). Its mutation affects the process of platelet release from megakaryocytes and may alter the structure of podocytes and cochlear epithelial cells 38. More than 40 mutations have been described and the mode of genetic inheritance is autosomal dominant 39.

The disease is mainly characterized by congenital macrothrombocytopenia, along with glomerulonephritis, sensorineural hearing loss, and cataract. In most cases, thrombocytopenia is usually mild, resulting in mild to moderate bleeding episodes in 25-50% of patients. Moreover, as platelet counts near the lower limit of normal have been described in a few individuals with MYH9-RD, large platelets are the only finding shared among all affected individuals 38 ^-^ 41. It is also common the presence of cytoplasmic inclusion in leukocytes, named Döhle corpuscles, which correspond to cytoplasmic aggregates of NMMHC-IIA. Hearing loss is the most frequent extra-hematological alteration, being reported in up to 60% of cases. Generally, it is progressive, bilateral, sensorineural, and can begin at any age. Cataracts occur in 16% of patients, with a mean age of presentation at 23 years of age 38 ^-^ 41.

Glomerulonephritis occurs in 30-70% of individuals. Proteinuria is the earliest manifestation, eventually causing nephrotic syndrome, associated or not with microscopic hematuria. In the majority of cases, progression to ESRD is usually rapid, requiring renal replacement therapy before the fourth decade of life. For a long time, due to the overlap of clinical manifestations, this disease was considered a variant of Alport syndrome. The presence of macrothrombocytopenia and the identification of the *MYH9* gene as the affected one allowed the recognition that these diseases are distinct disorders 41.

The diagnosis can be confirmed by immunofluorescence for NMMHC-IIA in neutrophils 42. Genotyping should also be performed whenever possible. Beyond further confirming the diagnosis, it may also provide important prognostic information due to genotype-phenotype correlation 43.

Renal biopsy is usually not indicated due to the risk of bleeding and non-specific histopathological characteristics, being reserved for cases in which a differential diagnosis with other glomerulopathy is necessary. The main histopathological findings include mesangial expansion and proliferation, and segmental glomerulosclerosis. Electron microscopy usually shows thickening of the glomerular basement membrane and effacement of the foot processes of podocytes 39.

Platelet transfusion is indicated in thrombocytopenic patients with active bleeding and as preoperative prophylaxis. Renin-angiotensin system blockade appears to be effective in reducing proteinuria and decreasing the progression of renal dysfunction 39.

Recently, we described the first case in Brazil of nephropathy associated with *MYH9* gene mutation in a 20-year-old male due to a *de novo* missense mutation in exon 1 of *MYH9* [c.287C>T; p.Ser(TCG)96(TTG)Leu]. The patient had nephrotic proteinuria and a progressive loss of renal function, with an annual decline in estimated glomerular filtration rate of 18 mL/min/1.73m^2^/year in the last 5 years. Unfortunately, he has not adhered to the use of ACE inhibitor at the onset of the disease, which may have blurred the potential benefit of renin-angiotensin-system blockade in the course of the disease 44.

### Fabry disease

Fabry disease is a rare X-linked lysosomal storage disorder caused by mutations in the *GLA* gene (position Xq22), which encodes the a-galactosidase A (a-GAL) enzyme. The mutation may lead to a total or a partial deficiency of the enzyme resulting in an inability to catabolize lipids with a-galactosyl terminal residues, mainly globotriaosylceramide (GB3). These, in turn, accumulate in the form of lysosomal deposits leading to cellular dysfunction, such as in endothelial cells, neurons, cardiomyocytes and renal cells, and ultimately to degenerative processes (fibrosis) and loss of function in different organs 45. Thus, Fabry disease is a multisystemic disorder with a wide spectrum of manifestations.

More than 1000 mutations in the *GLA* gene have been described (Human Genome Mutation Database). Not all of them are considered pathogenic, though. Each mutation is particular to a single family and depending on its type different levels of residual enzyme activity may be present. This may help, at least partially, to explain the great variability of manifestations and differences in the clinical course of the disease 45. Interestingly, an intrafamilial variability in the clinical manifestation of Fabry disease has been recognized. Moreover, it should be underlined that heterozygous women may also develop Fabry disease due to the skewed X-chromosome inactivation. Heterozygous women have a wider spectrum of presentation varying from asymptomatic carriers to severe clinical manifestation, as commonly seen in males 46 ^,^ 47.

The prevalence of Fabry disease ranges from 1:40,000 to 1:117,000 48 ^,^ 49. However, considering most physicians are not aware of the disease, clinical manifestations may be subtle, particularly in late-onset phenotypes, and that screening studies hardly include females it is plausible to suppose that its prevalence may have been underestimated. Among high-risk populations, such as patients with idiopathic hypertrophic cardiomyopathy, cryptogenic stroke or ESRD of undetermined etiology, the prevalence of Fabry disease has been reported to be higher 50. In the group of ESRD patients, it has been described to be as low as 0.04% until up to 1.16% in male dialysis patients 51 ^-^ 54. Screening studies in newborn males have reported an incidence of 1:3,100 55.

Two phenotypic presentations of Fabry disease have been recognized: the classical and the late-onset phenotypes. In the classical form, clinical manifestations, such as achroparesthesia, neuropathic pain, “Fabry crises”, angiokeratomas, hypo or hyperhidrosis, cochleovestibular and gastrointestinal disorders, generally begin during childhood or adolescence. Renal, cardiac, and cerebrovascular complications usually appear after the second decade of life 56. Cardiac manifestations include hypertrophic cardiomyopathy, predominantly of the left ventricle and of the interventricular septum, conduction disorders, which may lead to increased susceptibility to arrhythmias, and infarction 57. Common central nervous system manifestations are white matter lesion and ischemic stroke.

Renal impairment is characterized by the development of proteinuria, most often below the nephrotic range, and progressive loss of renal function. Importantly, proteinuria greater than 1g/24h and more than 50% of sclerotic glomeruli have been associated to poorer prognosis 58. Other renal manifestations comprise defect of urinary concentration (isosthenuria), distal renal tubular acidosis and parapyelic cysts. The progression to ESRD requiring RRT occurs around the 4th to 5th decades of life 59 ^,^ 60. Other clinical manifestations observed in Fabry disease patients are hearing loss, cold intolerance, intolerance to physical activity, and obstructive pulmonary disease. In the late-onset phenotypes one sole organ, the heart or the kidneys, is affected whereas the classic symptoms are usually absent or appear later in life 60 ^-^ 62.

Cerebrovascular and cardiovascular complications are currently the main causes of death in Fabry disease patients, occurring around the 5th to 6th decades of life. Fabry disease significantly impairs the quality of life and productivity of the individual, being associated with greater morbidity and lower survival 63.

The diagnosis of Fabry disease is generally suspected on the basis of a low a-GAL enzyme activity, which can be measured in plasma, leukocytes or dried blood spot (DBS). It is highly recommended performing molecular testing in every patient with low a-GAL enzymatic activity (i) to identify the specific mutation of the GLA gene 64 ^,^ 65 and (ii) to rule out non-pathogenic mutations (polymorphisms), such as the D313Y, that may cause enzymatic pseudo-deficiency and, consequently, lead to misdiagnosis of Fabry disease 65 ^,^ 66. Otherwise, one should be aware that the pathogenicity of certain variants, such as D313Y and R118C, is still a zone of uncertainty in Fabry disease. Reports have described carriers of these mutations with clinical manifestations of Fabry disease 67. Genetic, epigenetic, and environmental factors may influence the clinical presentation and severity of the diseases which might render the variant pathogenic in some but not in others. Obviously, this variability cannot be thoroughly detected by genotyping. Confirming the diagnosis of Fabry disease is a huge challenge and a matter of ascertainment. It should not rely on a single diagnostic tool alone, but requires a multifaceted approach integrating detailed clinical evaluation, detection of metabolic alterations (enzymatic activity of a-GAL, plasma or tissue accumulation of GB3) and genotyping. Diagnosing Fabry disease in females is particularly puzzling and molecular testing should always be performed regardless of the enzymatic activity that is greatly variable due to skewed X-chromosome inactivation. Finally, in the presence of diagnostic uncertainty, for instance when a variant of uncertain significance is detected, it may be necessary to perform a biopsy of an affected organ to confirm the diagnosis of Fabry disease by demonstrating the presence of lysosomal deposits 64 ^,^ 65.

**Figure 2:**
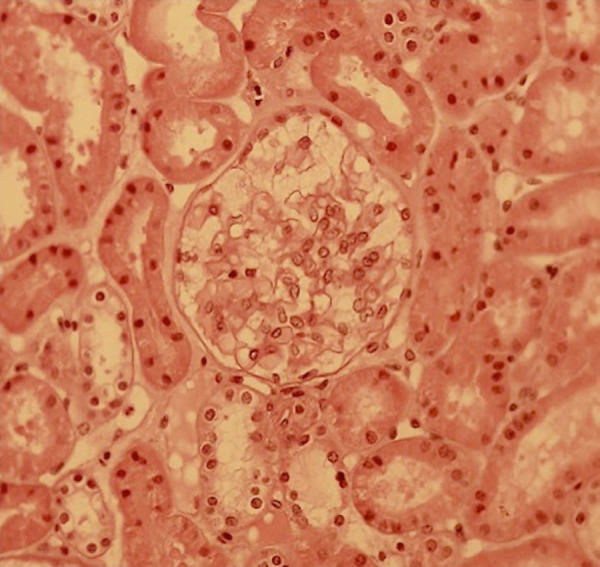
Vacuolization of podocytes in a kidney biopsy from a Fabry disease patient ( HE staining, magnification 40x).

Despite of being an invasive procedure, renal biopsy may be a helpful tool to both confirm the diagnosis and evaluate the efficacy of the therapy 68. One of the main renal histological findings in Fabry disease is the vacuolization of the renal cells, especially of podocytes, by light microscopy in sections stained by hematoxylin-eosin (Figure 2). This vacuolization corresponds to the enlarged secondary lysosomes packed with lamellated membrane structures, called zebroid bodies, observed by electron microscopy. The effacement of the foot process of podocytes is also a common, though non-specific, finding. Semi-thin sections stained with toluidine blue allows the visualization of the GB3 inclusions by light microscopy. Recently, it was proposed a score to standardize the report of the histological alterations in Fabry nephropathy 69. It is important to bear in mind that some medications, such as amiodarone and chloroquine, can lead to the formation of tissue lipid deposits, mimicking Fabry disease, in different organs, such as the kidneys and cornea 70.

There is not yet a reliable biomarker for the diagnosis, follow-up, and to monitor therapeutic response in Fabry disease patients. Serum and/or urinary levels of GB3 and of Lyso-GB3 have been proposed, being reported a superiority of the latter over the former 71. It has been suggested that podocyturia may be an early marker of renal damage, appearing before albuminuria, in Fabry nephropathy 72.

Before the advent of enzyme replacement therapy (ERT), the treatment of Fabry disease was only supportive and symptomatic. ERT, approved in 2001, was the first specific treatment for Fabry disease. Pivotal studies suggest that ERT is safe and effective, being able to modify the natural history of the disease 73 ^-^ 75. There are two different recombinant enzyme preparations available in the market: (i) agalsidase-alfa and (ii) agalsidase-beta. In general, current guidelines recommend ERT for all men over the age of 16, when there are signs or symptoms of the disease in children under 16 and in women with symptoms or with involvement of a noble organ 76. More recently, the use of a pharmacological chaperone as monotherapy for Fabry disease has been approved. Unlike the enzymatic preparations, it is given orally every other day. It may be used only in patients with amenable missense mutations and with a GFR greater than 30 mL/min/1.73m^2^ 77. A phase III clinical trial (NCT03180840) with a new replacement enzyme, Pegunigalsidase alfa, which runs in its superiority over the other two currently available enzyme formulations, is undergoing. As a new therapeutic approach for the future, pre-clinical studies have reported promising results of a substrate reduction therapy (Genz-682452) in association with ERT 78.

At last, but not least, it is worth mentioning that nephrologists play an important role in Fabry disease. Given that ESRD patients are considered a high-risk population, growing efforts have been made for screening Fabry disease in dialysis and transplant center. This screening strategy may lead to the identification of index cases together with at-risk family members. If in one hand this approach is crucial to detect a commonly overlooked disease and to potentially allow earlier initiation of treatment of relatives, on the other hand, physicians should be aware it may increase the number of doubtful cases and misdiagnosis due to the detection of variants of unknown significance.

## Conclusions

Hereditary rare kidney diseases represent a great challenge in Nephrology practice. In recent years, there has been a shift of paradigm in this field. Greater access to genetic testing and the increasing possibility of therapeutic interventions has fueled the interest in this area. Importantly, the investigation of regional differences in terms of prevalence of the different causes of CKD would be important and the findings could provide some guidance for genetic investigation. Spreading the interest on rare kidney diseases is pivotal to changing patient pilgrimage through several medical consultations until the correct diagnosis, unnecessary laboratorial exams, and ineffective therapeutic measures. On the other side, the medical community should be prepared to deal with rare kidney diseases patients care in order to avoid misdiagnosis and erroneous indication of costly therapy, when available.
